# PReS-FINAL-2030: Treatment with leflunomide results in a higher flare rate of chronic uveitis compared to methotrexate in patients with juvenile idiopathic arthritis treated with both drugs

**DOI:** 10.1186/1546-0096-11-S2-P43

**Published:** 2013-12-05

**Authors:** J Bichler, M Krumrey-Langkammerer, JP Haas, B Hugle

**Affiliations:** 1 German Center for Pediatric and Adolescent Rheumatology, Garmisch-Partenkirchen, Germany

## Introduction

Chronic anterior uveitis is a common complication of juvenile idiopathic arthritis (JIA). Leflunomide is a frequently used alternative to methotrexate in the treatment of joint manifestations of JIA. However, very little is known on the effect of leflunomide treatment on concurrent chronic uveitis in JIA.

## Objectives

To investigate patients with juvenile idiopathic arthritis with a history of non-simultaneous treatment of methotrexate and leflunomide, and to compare flare rates of uveitis during treatment periods with both drugs in these patients.

## Methods

The database of the German Center for Pediatric and Adolescent Rheumatology was searched for all patients admitted from January 2010 until October 2011 with a diagnosis of juvenile idiopathic arthritis and chronic anterior uveitis and treatment periods with both leflunomide and methotrexate. Patients with uveitis due to other causes were excluded. A retrospective chart survey was used to extract demographic data, diagnosis, and start and end times of treatment with leflunomide and methotrexate, respectively, concomitant medications and numbers of anterior uveitis flares. Anterior uveitis flare was defined as detection of any anterior chamber cells or flares after previously documented inactivity by an ophthalmologist. A generalized linear mixed model was constructed using a negative binomial distribution. Number of flares was used as the dependent variable, and the two treatments, LFN and MTX, were considered repeated measurements. The logarithm of time was used as an offset variable.

## Results

15 patients were included in the study, 9 patients with extended oligoarthritis, three with seronegative polyarthritis, two with persistent oligoarthritis and one with psoriatic arthritis. 100% had positive antinuclear antibodies. All patients were treated with methotrexate prior to leflunomide treatment, six patients had a second course of methotrexate and one patient a second course of leflunomide. 10 patients showed uveitis prior to treatment, and five patients developed uveitis on treatment with methotrexate. Median time of treatment with methotrexate was 51 months (range 26 - 167 months), and with leflunomide was 12 months (range 4 - 47 months). While on methotrexate, one patients each received etanercept and adalimumab, and one subsequent courses of both, compared to five patients on adalimumab and one each on etanercept and on infliximab while on leflunomide. On 1012 months of methotrexate treatment, 25 flares occurred, while on 247 months of leflunomide treatment, 15 flares occurred. This corresponds to a flare rate of 0.0247 flares/month on methotrexate treatment and 0.0607 flares/month on leflunomide treatment (p = 0.008).

## Conclusion

Despite more co-medications potentially improving the uveitis outcome in leflunomide treatment periods, patients showed significantly more flares of uveitis compared to treatment periods with methotrexate. Further research is necessary to assess leflunomide efficacy in chronic uveitis associated with JIA.

## Disclosure of interest

None declared.

